# Use of Non-Pharmacological Supplementations in Children and Adolescents with Attention Deficit/Hyperactivity Disorder: A Critical Review

**DOI:** 10.3390/nu12061573

**Published:** 2020-05-28

**Authors:** Eleonora Rosi, Silvia Grazioli, Filippo Maria Villa, Maddalena Mauri, Erica Gazzola, Marco Pozzi, Massimo Molteni, Maria Nobile

**Affiliations:** 1 Child Psychopathology Unit, Scientific Institute, IRCCS Eugenio Medea, 23842 Bosisio Parini, Italy; eleonora.rosi@lanostrafamiglia.it (E.R.); filippomaria.villa@lanostrafamiglia.it (F.M.V.); maddalena.mauri@lanostrafamiglia.it (M.M.); massimo.molteni@lanostrafamiglia.it (M.M.); maria.nobile@lanostrafamiglia.it (M.N.); 2 PhD Program in Neuroscience, School of Medicine and Surgery, University of Milano-Bicocca, 20126 Milan, Italy; 3 Department of Psychology, University of Milano Bicocca, 20126 Milan, Italy; e.gazzola1@campus.unimib.it; 4 Scientific Institute, IRCCS Eugenio Medea, 23842 Bosisio Parini, Italy; marco.pozzi@lanostrafamiglia.it

**Keywords:** dietary supplementations, non-pharmacological treatment, rehabilitation, neurodevelopment, attention deficit hyperactivity disorder (ADHD), children, adolescents

## Abstract

Attention deficit hyperactivity disorder (ADHD) is a common neurodevelopmental disorder in children and adolescents, with environmental and biological causal influences. Pharmacological medication is the first choice in ADHD treatment; recently, many studies have concentrated on dietary supplementation approaches to address nutritional deficiencies, to which part of non-responses to medications have been imputed. This review aims to evaluate the efficacy of non-pharmacological supplementations in children or adolescents with ADHD. We reviewed 42 randomized controlled trials comprised of the following supplementation categories: polyunsaturated fatty acids (PUFAs), peptides and amino acids derivatives, single micronutrients, micronutrients mix, plant extracts and herbal supplementations, and probiotics. The reviewed studies applied heterogeneous methodologies, thus making it arduous to depict a systematic overview. No clear effect on single cognitive, affective, or behavioral domain was found for any supplementation category. Studies on PUFAs and micronutrients found symptomatology improvements. Peptides and amino acids derivatives, plant extracts, herbal supplementation, and probiotics represent innovative research fields and preliminary results may be promising. In conclusion, such findings, if confirmed through future research, should represent evidence for the efficacy of dietary supplementation as a support to standard pharmacological and psychological therapies in children and adolescents with ADHD.

## 1. Introduction

Attention deficit hyperactivity disorder (ADHD) is a neurodevelopmental disorder affecting about 5% of children and 2.5% of adults worldwide. It is characterized by dysregulated cognition and behaviors, resulting in inattention, excessive motor activity, and impulsivity [1].

Drug treatments for ADHD mainly act to potentiate the action of catecholamines, which are neurotransmitters involved in the prefrontal cortex responsible for the maintenance of attention and cognitive control [2].

Stimulants, the first choice for ADHD drug treatment, include methylphenidate that inhibits dopamine reuptake and amphetamines that inhibit dopamine and noradrenaline reuptake and increase dopamine release. Atomoxetine is the most common non-stimulant medication for ADHD and acts by inhibiting norepinephrine reuptake [3].

The efficacy of stimulants and non-stimulants in reducing ADHD symptoms is well documented in children and adults [4,5]. However, the tolerability of these drugs can be scarce in some patients, warranting treatment interruption. At least 10–30% of children with ADHD may not benefit from these medications due to non-response or adverse effects [6,7] such as decreased appetite, insomnia, stomachache, headache, weight loss (potentially leading to growth retardation), tics, increases in blood pressure, and potential abuse or misuse [8]. Although less effective than stimulants, non-stimulant medication are first choice treatments for individuals with co-diagnoses including tic, mood, and anxiety disorders [3]. However, atomoxetine use has been associated with increased risk of suicidal behavior in youths [9].

ADHD is a multifactorial disorder, in which genetic and biological factors have important roles; ADHD symptomatology expression is also influenced by environmental factors, like nutrition [10]. To explain the role of nutritional deficiencies, to which part of non-responses to medications have been imputed, several studies focused on supplementation approaches, as alternative or additional therapies.

Within this research area, many studies have focused on macronutrient supplementations, mainly polyunsaturated fatty acids (PUFAs), that are precursors of eicosanoids and are relevant components of cell membranes and of phospholipids. PUFAs influence the quality of growth and development [11]. Research about PUFAs adjunctive treatments for ADHD did not produce convincing evidence, probably due to the heterogeneity of methods and doses administered [12,13].

Few studies have also examined the role of other macronutrients such as peptides and amino acids derivatives, which can act as metabolic adjuvants. In particular, there are experimental trials using Acetyl-L-carnitine (ALC) to optimize mitochondrial fatty acid oxidation. It has been reported that ALC treatment increased sodium levels in the cingulate cortex and the 5HIAA/5HT ratio in both cingulate cortex and medial frontal cortex in adolescent impulsive rats [14] and it could have a link with hyperactivity and impulsivity symptoms in children with ADHD [15]. L-carnosine (a dipeptide composed of β-alanine and l-histidine) is known as an antiaging antioxidant and neuroprotective compound, and it is found highly concentrated in brain tissues [16]. L-carnosine has also been associated to ADHD symptoms [17]: it accumulates in the subfrontal cortex and may enhance frontal lobe functions [18], interesting for ADHD research. Lastly, Phosphatidylserine (PS), a naturally occurring phospholipid, modulates the activity of receptors, enzymes, ion channels and signaling molecules, and contributes to regulate membrane fluidity [19]. PS has been shown to counteract the stress-induced activation of the hypothalamic-pituitary-adrenal axis [20]; it has been involved in ADHD symptomatology and short-term auditory memory [21].

Other studies have investigated the role of various micronutrients (e.g., oligoelements, vitamins, minerals) as alternative or supporting non-pharmacological treatment for ADHD.

Vitamin D deficiency is relevant in the pathogenesis of psychiatric disorders including autism, depression, schizophrenia, and ADHD [22,23,24]. Vitamin D can act on central dopamine levels via different mechanisms, including calcium transition, antioxidant properties and gene expression [25].

Another micronutrient linked to dopamine metabolism is zinc, a cofactor of many metalloenzymes and metal–enzyme complexes [26]. Studies conducted on animals and humans associated zinc deficiency and hyperactivity [27,28]. Researchers also focused on broad-spectrum supplementation approaches, highlighting the efficacy of minerals and vitamins combinations [29].

Complementary or alternative medicine approaches propose herbal extracts for children with ADHD. However, these studies have not yet produced structured evidence [30].

Lastly, an interesting hypothesis is that gut microbiota and probiotics could influence brain activity and behaviors and psychiatric symptoms [31]. In rodent studies, modifications of gut microbiota are associated with cerebral signals modification, in cortical and subcortical regions, linked to affective and sensory functions [32]. Evidence in humans is preliminary; however, chronic intake of probiotics is associated in healthy adults with altered brain connectivity during affective and attentive tasks [32].

Given the number and heterogeneity of studies conducted on nutritional supplementation in ADHD, the present review aims at offering a systematization for the results of studies conducted from January 2010 to March 2020 and evaluating the efficacy of non-pharmacological dietary supplementations in ADHD.

We defined the present review as “critical” to highlight our aim to offer clinicians an analytical and up-to-date point of view in the clinical management of children and adolescents with ADHD, specifically in a nutritional supplementation framework alone or in combination with pharmacological treatment. Moreover, this review did not have a systematic or quantitative objective.

## 2. Materials and Methods

The present review is reported according to the preferred reporting items for systematic reviews and meta-analyses (PRISMA) [33]. We searched PubMed from January 2010 to March 2020 using the following string: (probiotic OR prebiotic OR vitamin OR mineral OR phytonutrient OR amino acid OR supplementation OR non-pharmacological) AND (ADHD OR attention deficit hyperactivity disorder) NOT review [All Fields] NOT adult NOT infant. The search manually completed with relevant articles. We included randomized controlled trials involving children and adolescents with ADHD receiving non-pharmacological supplementation. We focused on studies addressing cognitive or behavioral outcomes; we excluded papers not written in English, researches addressing other diagnoses than ADHD, studies with no administration of non-pharmacological supplementations, studies that did not report behavioral/cognitive outcomes, and studies on preschool or adult subjects. Specifically, our search did not include infants (<5 years) given ADHD onset which typically corresponds to school age [1]. Moreover, since this review aim was to offer evidences regarding clinical management of children and adolescents with ADHD, we decided to exclude adults in the PubMed search.

## 3. Results

**Figure 1:**
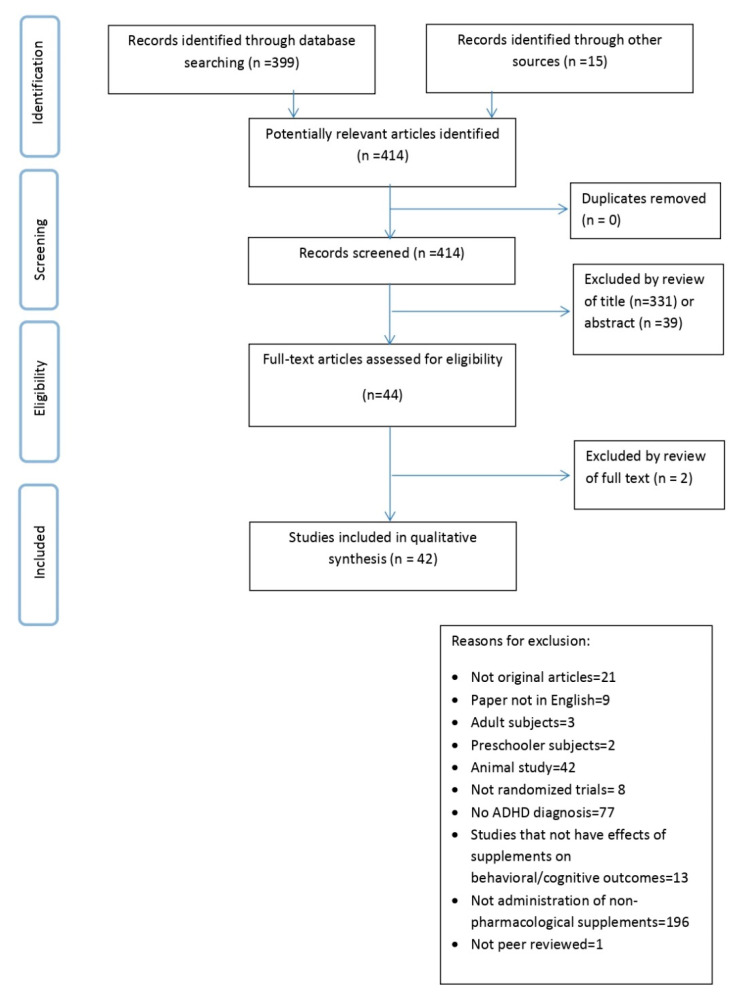
Preferred reporting items for systematic reviews and meta-analyses (PRISMA).Flow diagram of the study selection process.

Our search found 414 articles. After title and abstract screening, articles retained were 44; we excluded two articles after full text review. In total, 42 articles are included in the present review. The PRISMA flow chart is shown in Figure 1.

In the following text and tables, results are presented according to supplementation categories as follows:

- PUFAs;
- peptides and amino acids derivatives;
- single micronutrient (Zinc or Vitamin D);
- micronutrients mix;
- plant extracts or herbal supplementations;
- probiotics.

### 3.1. PUFAs

#### 3.1.1. Methodologies

In total, 20 studies focused on supplementation with PUFAs [34,35,36,37,38,39,40,41,42,43,44,45,46,47,48,49,50,51,52,53] (see Table 1a): fourteen were on patients without comorbidities [34,36,37,38,40,41,42,43,44,45,46,47,50,53], two with oppositional defiant disorder (ODD) [35,39], and four with various co-diagnoses, such as specific learning disorder, ODD, conduct disorder, tic disorder, anxiety, Tourette syndrome, or psychomotor difficulties [48,49,51,52]. Two researches [42,43] include very small samples.

These studies supplemented with various omega-3 fatty acids. In total, 18 studies provided docosahexaenoic acid (DHA) and eicosapentaenoic acid (EPA) [34,35,36,37,38,39,40,41,42,44,45,46,47,48,49,50,52,53], one study provided alpha linolenic acid (ALA) [43], and one study did not specify which omega-3 was used. Three studies supplemented omega-6: one used gamma-linolenic acid (GLA) [36], while two did not specify which omega-6 was used [35,51]. One study provided, together with EPA and DHA, conjugated linoleic acid (CLA), a precursor of a distinct family of PUFAs (both omega-3 and omega-6) [47]. In two linked studies, authors prescribed linoleic acid (LA) omega-6 as a control condition compared to two omega-3 groups [48,49]. Seven studies added vitamins to EPA and DHA [40,44,47,48,49,52,53]; each of the seven provided vitamin E, whereas in Cornu et al. added vitamins A and D [40]. Six studies allowed pharmacotherapy concomitant with PUFA supplementation [34,35,36,37,50,52], while two added to EPA and DHA supplementation with PS [46] or docosapentaenoic acid [52]. Although PUFAs represent now the most used supplementation in this field, there is too much heterogeneity around the specific PUFAs used. It remains unclear if the efficacy of results depend on individual type of omega-3/6 or the whole of macronutrients.

Randomization between treatment and placebo groups is equal, except for one study [46], but there is still too much heterogeneity around the number of treatment groups.

Concerning outcomes, several studies used both multiple questionnaires/ratings [36,37,41,44,46] and cognitive tasks [38,39,40,42,43,48,49,52,53] and only one includes fMRI assessment [38]. However, other many studies kept in consideration only one questionnaire [34,35,45,47,50,51], as a result future researches could integrate better outcome measures.

#### 3.1.2. Results

Regarding results, 13 studies highlighted various improvements related to PUFAs supplementation [35,36,38,39,41,44,45,46,48,49,51,52,53]. Six of those used EPA and DHA combinations [38,39,41,45,48,49], three used unspecified omega3 + omega6 combinations [35,36,51], three added vitamin E to the omega3 formula [44,52,53], and one study prescribed PS together with omega3 [46]. In these studies, ameliorations were not related to specific cognitive or behavioral domains. The remaining seven studies [34,40,42,43,47,49,50] did not find positive results linked to PUFAs supplementation. Future investigations with homogeneous methodologies are needed to clarify the reason of non-significant results or could better specify the role of PUFAs supplementation on ADHD symptoms.

### 3.2. Peptides and Amino Acids Derivatives

#### 3.2.1. Methodologies

We identified three studies [15,17,21] using Acetyl-L-carnitine (ALC), l-carnosine, and PS (Table 1b). It is not possible to depict a systematic comparison of those studies, given the heterogeneous supplementation approaches. Specifically, two works [15,17] compared subjects who tok a placebo to a group that took supplementation as an add-on to MPH. On the contrary, one study [21] used PS alone in the active treatment group and placebo in control group. All studies comprised uniform samples, except Ghajar et al. [17], who also included children with ODD and applied equal randomization in treatment and placebo groups. Two studies [15,17] considered similar outcome measures, including parent and teacher questionnaires regarding children’s behavioral and cognitive symptoms. Hirayama et al. [21] used a go/no-go task and ADHD diagnostic criteria. Although these studies focused on different supplementations, they applied similar methodologies, especially regarding sample, randomization, and outcomes. The use of neuropsychological and physiological parameters could be improved and integrated to better understand response to treatment mechanisms. Moreover, future researches could address PS efficacy as an add-on to MPH.

#### 3.2.2. Results

Regarding these studies results, ALC alone did not improve ADHD symptoms [15], but it reduced adverse effects when taken in addition to MPH. Moreover, l-carnosine seemed to improve behavioral problems according to parents [17]. PS had effects on ADHD symptoms, short-term auditory memory, and cognitive tasks [21]. In conclusion, l-carnosine and PS seem to have some effects on ADHD symptoms or cognitive domains but it remains unclear their specific role and mechanisms of action.

### 3.3. Single Micronutrient (Zinc or Vitamin D)

#### 3.3.1. Methodologies

Some studies tested zinc [54,55,56] or vitamin D [25,57,58] (Table 1c). Zinc was used as sulfate [55,56] or glycinate [54] and vitamin D as generic [25] or D3 [57,58]. All studies provided micronutrients in addition to MPH [25,55,56,57,58] or dextroamphetamine [54]. Moreover, only Arnold et al. [54] compared the effects of micronutrients (and no pharmacological treatment) with placebo. All studies except two [54,58] included patients without comorbidity. Each study applied equal randomization between groups, even if the number of subjects was low [e.g., 58]. These works used similar methodologies, but several studies [25,55,57,58] used only parent-ratings as outcome measure.

#### 3.3.2. Results

A link between zinc consumption and attention improvement was found in two studies [55,56]: zinc treatment improved inattention scores on parent questionnaires [55] and had effects on attention deficit disorder subtype of ADHD [56]. One study [54] did not find significant differences between zinc supplementation and placebo in the outcome measures; however, children taking zinc supplementation in addition to dextroamphetamine showed better drug dose optimization. All studies regarding vitamin D supplementation highlighted positive outcomes on parent-rated behavioral indexes or ADHD symptoms [25,57,58].

### 3.4. Micronutrients Mix

#### 3.4.1. Methodologies

Several studies focused on broad combinations of vitamins and minerals [59,60,61,62,63] (Table 1d). One used a mixture of vitamin D and magnesium [60], two provided “daily essential nutrient formula”, which contained 13 vitamins, 17 minerals, and 4 amino acids [59,61]. The remaining studies represent further analyses [63] and follow-up research [62] of the Rucklidge et al. paper [61]. All studies were comprised of children with several comorbidities and applied equal randomization between groups. All studies used multiple ratings, except for Hemamy et al. [60], and one work using magnetic resonance imaging (MRI) data as an outcome (however, this last did not find any significant effect) [59].

#### 3.4.2. Results

These studies highlighted improvements in several behavioral, emotional, and cognitive ADHD symptoms. Furthermore, the follow-up study [62] offers additional evidences regarding possible efficacy of micronutrients mix. However, these studies are characterized by low numerosity and high heterogeneity of samples and treatments.

### 3.5. Plant Extracts or Herbal Supplementations

#### 3.5.1. Methodologies

Seven studies focused on plant or herbal extracts containing a mix of micronutrients, vitamins, and macronutrients (Table 1e). It is not possible to depict a systematic comparison of those studies, given the heterogeneous supplementation approaches. Specifically, one work used Ginkgo biloba plant extract as an add-on to MPH [69]. Others examined the efficacy of an herbal compound [64], Korean red ginseng (KRG) [65] or tocotrienol-rich fractions [70], as a single treatment compared to placebo, ningdong granule [66], sweet almond syrup [67], or Ginkgo biloba [68], compared to MPH. These studies focused on patients with ADHD symptoms without comorbidities and they applied an equal randomization, except for one work [64]. Each study used multiple ratings as outcomes, one of which [65] considered neurophysiological assessment and another cognitive tasks measures [64].

#### 3.5.2. Results

All but two studies [68,70] found beneficial effects related to supplementation, in terms of improved symptomatology and/or less adverse effects as compared to MPH. Improvement in attention indexes was found in studies using a blend of herbs [64], Korean red ginseng [65], and Ginkgo biloba as an add-on to MPH [69]. Parent-, teacher-, or clinician-rated behavioral improvement was reported with KRG [65], ningdong granule [66], sweet almond syrup [67], and Ginkgo biloba as an add-on to MPH [69]. No adverse effects of supplementations were highlighted except for one study that reported increased appetite with sweet almond syrup [67], whereas half of the studies reported adverse effects of MPH [66,67,68]. One study [65] showed that KRG reduced the electroencephalography theta/beta ratio, a marker of cognitive processing capacity, significantly more than placebo. These results are heterogeneous and preliminary, and thus future homogeneous investigations that consider physiological parameters could offer more systematic evidences regarding herbal or extract supplementations. Furthermore, conflicting results [e.g., 68–69] between identical supplementation may be due to the different objectives: in one case authors aimed at comparing effects of supplementation with MPH [68], in the other case effects of MPH and non-pharmacological treatment together were compared to placebo [69].

### 3.6. Probiotics: Methodologies and Results

Only one paper focused on probiotics supplementation, through a different study design compared to the other reviewed studies [71] (Table 1f). A six-months-lasting probiotic supplementation was administered soon after childbirth and a follow-up assessment was conducted after 13 years. The quantity of Bifidobacterium species bacteria in the feces of children later diagnosed with ADHD or Asperger syndrome was found to be lower as compared to healthy children. ADHD or Asperger syndrome was diagnosed in 6/35 (17.1%) children in the placebo and none in the probiotic group (*p* = 0.008). This last study offers preliminary suggestions regarding probiotics supplementation as a preventive treatment, however further randomized clinical trials are needed to offer more systematic evidence regarding this treatment efficacy.

**Figure 2:**
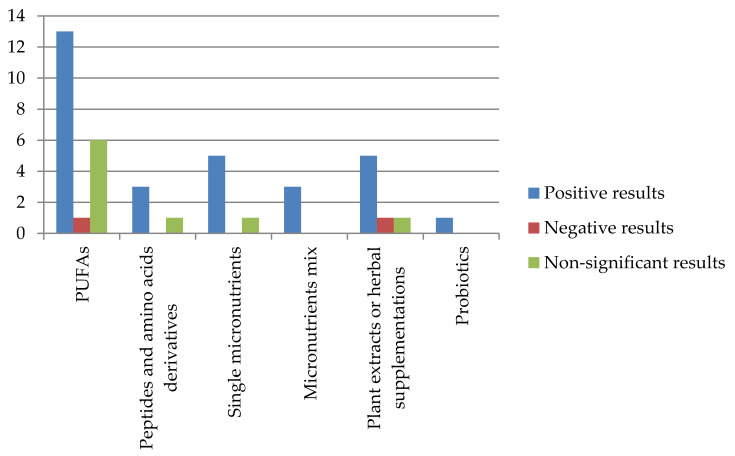
Effect of supplementations in ADHD symptoms.

Figure 2 summarizes the main findings reported by the included works regarding nutritional supplementations on ADHD behavioral or cognitive symptoms; the vertical axis indicates the number of studies.

## 4. Discussion

We investigated the recent literature about the efficacy of non-pharmacological treatments for ADHD in children and adolescents, alone or in combination with pharmacological treatment.

### 4.1. Discussion of Methodologies

It is relevant that nearly half of the reviewed studies used supplementation with PUFA (mostly EPA and DHA as omega3 PUFAs and, for some, omega6; see Table 1a). This supplementation approach could be linked to previous evidence suggesting the involvement of lower blood levels of DHA in children and adolescents with ADHD [12].

The other reviewed studies addressed the efficacy of peptides and amino acids derivatives (Table 1b), micronutrients (alone or in combination; see Table 1c,d), and plant or herbal extracts (Table 1e); one study investigated the association between early-life probiotics supplementation and ADHD or Asperger syndrome diagnoses at puberty (Table 1f). These supplementation approaches are less frequently reported in the scientific literature as compared to PUFA supplementation.

Each reviewed study used different combinations and doses of drugs and/or non-pharmacological supplementations. Therefore, it is not possible to draw systematic conclusions on optimal type or dose of compound that could be useful in the treatment of ADHD symptomatology.

Regarding outcome measures, Table 2 depicts a summary of sources of information that were considered in the reviewed studies. Table 2 highlights the need to consider homogeneous outcome variables in future research to obtain more systematic evidence related to the same outcomes. Moreover, objective neurophysiological outcomes should be more consistently evaluated together with clinical evidence.

### 4.2. Discussion of Results

The majority of reviewed papers reported improvements but no specific effect of different supplements was found, thus suggesting a non-specific beneficial influence of micro- and macro-nutrients on a broad spectrum of functions and symptoms. A possible explanation of this result could be ascribed to general environmental and dietary influences that have been previously associated to the severity of ADHD symptoms in children and adolescents, such as low socioeconomic status, parents’ education, and unhealthy diet [72,73]. In this framework, it is still unclear whether ADHD onset and persistence over time represent the cause or the effect of unhealthy dietary patterns that could lead to nutritional deficits [72,73]. In any case, this review suggests that non-pharmacological supplementation, prescribed on the basis of individual nutritional deficiencies, could constitute a valid clinical path. It is not clear whether supplementation has a role for patients with no dietary imbalance. Moreover, the substances that are contained in various supplementations could benefit brain functioning but may also influence overall physiological functioning in children and adolescents, given a non-specific effect of these compounds. Clinicians should support alternative or additional treatment options only after appropriate blood tests and medical examinations.

In any case, the supplementation approach seems to be valid in combination with pharmacological treatment, as highlighted by positive results of MPH combination with PUFAs [35,36,37,52,56], peptides or amino acid derivatives [15,17], zinc [55,56], vitamin D [25,57,58], vitamin D and magnesium [60], and sweet almond syrup [67]. In these studies, ameliorations were found in behavioral symptoms as reported by parents and clinicians, together with less adverse events compared to pharmacotherapy alone. Hence, drugs and ad hoc nutritional supplementation could represent a valid therapeutic approach.

Other studies focused on children and adolescents who were not under pharmacological treatment for reasons including low compliance, adverse effects or non-response. This second group of studies found mixed results, in terms of finding beneficial effects of supplementation alone and of finding no effect at all. However, the majority of these studies reported a beneficial effect of supplements over placebo.

Specifically, 8 out of 14 studies regarding PUFA supplementation alone found symptoms amelioration over placebo in attention, psychosocial functioning, emotional problems, behavior as reported by parents and teachers, and working memory [38,39,41,44,45,46,51,53]. The only study addressing phosphatidylserine supplementation found positive effects of treatment over placebo in behavioral and cognitive symptomatology as reported by clinicians and through a go/no-go computerized task [21]. However, one study prescribed zinc supplementation against placebo and found no improvement in behavior, memory, or attention [54]. Five studies used plant or herbal extracts versus placebo. Two found significant beneficial effects of a patented blend of herbs (compound herbal preparation) and Korean red ginseng on attention and symptomatology as reported by clinicians [64,65]. Two studies found similar effects of ningdong granule or sweet almond syrup as compared to MPH treatment in behavioral measures reported by parents and teachers, with fewer side effects related to herbal supplementation than MPH [66,67]. Lastly, one study reported greater parent- and teacher-rated behavioral amelioration effects of MPH as compared to Ginkgo biloba supplementation alone [68] and another [70] did not find efficacy using tocotrienol-rich fractions compared to placebo. The only study concerning early-life probiotic supplementation revealed positive effects compared to placebo preventing ADHD onset later in life [71]. Studies regarding micronutrients mix supplementation found beneficial effects over placebo in general functioning, emotional dysregulation, aggression, and attention [59,61,62]. Importantly, a follow-up work by Rucklidge et al. identified various factors related to response to treatment with micronutrients mix, such as lower pre-treatment folate and B12 levels, being female, greater severity of symptoms and co-occurring disorders in pre-treatment condition, more pregnancy complications, and fewer birth problems [63]. This work highlighted the role of biological and environmental variables related to response to non-pharmacological treatment. This last area of research needs further research, given the high heterogeneity of results due to confounding biological and environmental variables.

## 5. Limitations

There were limitations within the articles described in this review. Results should be interpreted in the light of high heterogeneity related to various methodological factors. Indeed, the included works considered heterogeneous treatments, trial durations, methodologies (e.g., supplementation used as unique or combined treatment), and outcomes, even within the same category of supplements. Hence, it was not possible to carry out a meta-analysis of research results, which instead would be auspicable to provide clinicians with more systematic evidence. Moreover, samples were not uniformly involving only children with ADHD diagnoses; other comorbidities or typically developing children were included in some samples. Studies in this research field are also susceptible of cultural influences such as local dietary habits, thus making results difficult to generalize.

Although the majority of studies used similar parent and/or teacher assessment measures as primary outcomes, in many cases clinicians’ evaluation or neurophysiological / neuropsychological assessments were lacking. Only three studies [38,59,65] used neurophysiological data, like magnetic resonance imaging or electroencephalography. These kinds of assessments should be included in future research.

Lastly, the majority of studies found beneficial effects, but this may be due to the fact that only studies that found effects were published. However, a formal evaluation of bias was not conducted due to the non-systematic nature of this review.

## 6. Conclusions

This review suggest that supplementation approaches may be effective in add-on to pharmacotherapy in improving some behavioral and neuropsychological indicators in children and adolescents with ADHD. The heterogeneity of results suggests that supplementation should be personalized based on each patient’s dietary issues. Several supplementation components that are still poorly investigated and may be effective. Moreover, some nutritional supplementations could represent an alternative treatment or rehabilitation in situations of non-response or poor compliance or lack of tolerability of drug treatments, a field that must still be investigated further.

## Figures and Tables

**Table 1:** (**a**) Studies comprising supplementation with polyunsaturated fatty acids (PUFAs). (**b**) Studies comprising supplementation with peptides and amino acids derivatives. (**c**) Studies comprising supplementation with a single micronutrient. (**d**) Study comprising supplementation with micronutrients mix. (**e**) Studies comprising supplementation with plant or herbal extracts. (**f**) Study comprising supplementation with probiotics.

**(a)**					
**Authors, Year**	**Sample and Age**	**Methodology and Durations**	**Daily Doses**	**Outcome Measures**	**Main Results**
Anand et al., 2016 [34]	50 ADHD (35 males), no other neuropsychiatric comorbidities. Age range: 4–11 years (6 ± 2.1 years).	4 months DBRCT 25 subjects taking ATX+ PUFA. 25 subjects taking ATX.	ATX: 0.5 mg/kg/day. PUFA: EPA 180 mg + DHA 120 mg/day.	CPRS-R: parent-rated behavioral indexes.	CPRS-R: Non significant trend: supplementation group improved in ADHD scores as compared to the control group. Improvement was more evident, although not significant, in males with combined type of ADHD.
Assareh et al., 2017 [35]	40 ADHD (30 males), ODD was present in 21 subjects. Age range: 6–12 (PUFA group: 9 ± 2 years; Placebo group 9.2 ± 2 years).	10 weeks DBRCT Unspecified subjects taking MPH +DHA + EPA+ Omega 6. Unspecified subjects taking MPH + Placebo.	MPH: 0.3 mg/kg/day (in 2 doses). Increased to 1 mg/kg/day for 2 weeks. EPA:33 mg/day. DHA: 241 mg/day. Omega 6: 180 mg/day. Placebo: similar to other capsules.	Parent rated ADHD-RS every two weeks.	ADHD-RS: Significant time effect: both groups showed an improvement in symptomatology over time.
Barragán et al., 2017 [36]	90 ADHD (60 males), no other neuropsychiatric comorbidities. Age range: 6–12 years (8.27 ± 1.74 years).	12 months RPT 20 subjects taking MPH. 22 subjects taking Omega-3/6. 27 subjects taking MPH + Omega-3/6.	MPH: 0.3 mg/kg/ day, increased to 0.5 mg/kg/day after the first 2 weeks. The dose was increased to 1 mg/kg/day depending on response and tolerability. Omega-3/6: EPA: 558 mg\day. DHA: 174 mg\day. GLA: 60 mg\day.	Parent rated ADHD-RS. CGI-S: assessment of severity as reported by clinician and parents.	ADHD-RS and CGI-S: - Significant time x treatment effect: MPH + supplementation group showed greater improvements compared to supplementation alone. CGI-S: - Slow decrease in Omega-3/6 groups, compared with a rapid decrease and subsequent slight increase in the MPH-containing groups. - Adverse events were numerically less frequent with Omega-3/6 or MPH + Omega-3/6 than MPH alone.
Behdani et al., 2013 [37]	69 ADHD (55 males), no other neuropsychiatric comorbidities. Age range: 7–15 years (8.7 ± 1.7 years).	Eight weeks DBRCT 36 subjects taking MPH + Omega 3 33 subjects taking MPH + Placebo.	MPH: 2.5–5 mg/day. Increased 2.5–5 mg weekly, to attain a final dose of 1 mg/kg (in two or three doses). Omega-3:1000 mg\2 times a day (240 mg of DHA and 360 mg of EPA). Placebo: similar to Omega 3 capsules.	Parent and teacher rated ADHD-RS.	ADHD-RS: Significant time effect: reduction in both parent’s and teacher’s rating scores in both groups.
Bos et al., 2015 [38]	38 ADHD; 38 TD (76 males), no other neuropsychiatric comorbidities. Age range: 8–14 years (ADHD:10.3± 2.0 years; TD: 10.9 ± 2.0 years).	16 weeks DBRPCT 19 ADHD^*^ taking Omega 3. 19 ADHD^*^ taking Placebo. 20 TD taking Omega 3. 18 TD taking placebo. *subjects took MPH before trial.	Omega 3 fortified margarine: 10 g (650 mg DHA and 650 mg EPA).Placebo: 10 g of similar margarine.	CBCL (parent-rated) SWAN (parent-rated) fMRI with G/No-Go paradigm	CBCL: Significant main effect of treatment: supplementation group had less attention problems compared to placebo groups.
Pei-Chen Chang et al., 2019 [39]	92 ADHD (79 males), ODD was present in 51 subjects. Age range: 6–18 years (9.49 ± 3.05 years).	12 weeks DBRPCT 48 subjects taking PUFAs. 44 subjects taking Placebo.	PUFAs (EPA): 1.2 g/day. Placebo: 1.2 g/day soybean oil.	CPT: computerized continuous performance test to evaluate attention measures. Parent, teacher and self-rated SNAP IV: checklist of DSM-IV ADHD symptoms.	CPT: Significant time x treatment interaction effect: supplementation group showed greater improvement in focused attention compared to placebo group.
Cornu et al., 2018 [40]	162 ADHD (127 males), no other neuropsychiatric comorbidities. Age range 6–15 years (6.9 ± 2.9 years).	Three months DBRPCT 77 subjects taking Omega 3. 80 subjects taking Placebo.	Omega 3 for subjects aged 6–8: EPA 336 mg + DHA 84 mg/day. Omega 3 for subjects aged 9–11: EPA 504 mg + DHA 126 mg/day. Omega 3 for subjects aged 12–15: EPA 672 mg + DHA 168 mg/day. Supplementation capsules also contained 100 mg vitamin A + 1.25 mg vitamin D + 3.5 mg vitamin E. Placebo: similar to Omega 3.	Parent-rated ADHD-RS-IV: ADHD symptomatology as outlined in the DSM-IV-TR. CPRS-R: parent-rated behavioral indexes. “L’Alouette” test: french reading test. Attentional Performance Tests for Children. Children’s Depression Inventory: self-rated depression symptomatology.	ADHD-RS: the severity score decreased in both groups. The decrease was significantly higher in the placebo group.
Crippa et al., 2019 [41]	50 ADHD (46 males), no other neuropsychiatric comorbidities. Age range: 7–14 years (Omega 3 group: 11.06 ± 1.85 years; Placebo group: 10.91 ± 1.42 years).	Six months DBRPCT 25 subjects taking Omega 3. 25 subjects taking Placebo.	Omega 3: DHA 500 mg/day. Placebo: similar to Omega 3.	Parent-rated ADHD-RS version IV: ADHD symptomatology as outlined in the DSM-IV-TR. CPRS-R: parent-rated behavioral indexes. SDQ: parent-rated emotional and behavioral indexes. CHQ: parent-rated measure of quality of life. CGI and C-GAS: assessment, severity and improvement of symptoms as reported by clinician.	A main amelioration effect over time was found in the following measures: ADHD-RS, CPRS-R ADHD index, CPRS-R Global Index restless – impulsive, CPRS-R Global Index total, DSM-IV hyperactive– impulsive scale and DSM-IV total, SDQ Hyperactivity scale and on SDQ total difficulties score, CGI severity, C-GAS. Children in the DHA group showed significantly higher amelioration effect compared to the placebo group in the CHQ. Psychosocial summary and emotional problems on SDQ.
Dean et al., 2014 [42]	16 ADHD, 11 CD (17 males), no other neuropsychiatric comorbidities. Age range: 7–14 years (10.3 ± 2.2 years).	Six weeks RPCCT+ Follow-up 12 subjects taking fish oil capsules for six weeks (Phase 1) followed by placebo capsules for six weeks (Phase II). Nine subjects taking placebo capsules for six weeks (Phase I) followed by fish oil for six weeks (Phase II).	Fish oil: 4 g/day, (400 mg EPA + 2000 mg DHA). Placebo: 4 g\day (low polyphenol, olive oil and 10 mg standard fish oil to assist in maintaining blinding).	Children’s Aggression Scale – Parent Version. MOAS (parent-rated) SDQ (parent-rated) Family Assessment Device General Functioning Scale (FAD) ADHD-RS (parent-rated) Cognitive functioning: -Executive control trail-making task. -Response inhibition–stop signal task Cognitive control: -Eriksen flanker task.	Aggressive behaviour: No effect of fish oil treatment was observed on changes in total scores despite it increased serum concentrations of EPA and total omega-3 s. SDQ: Fish oil group worsened in Conduct Subscale but improved in Hyperactivity Subscale. No effect of fish oil supplementation was observed for other SDQ subscales or SDQ total score, the ADHD rating scale, or family functioning (FAD). Cognitive measures: Fish oil supplementation did not lead to any changes on the stop signal task, trail-making task, or flanker task.
Dubnov-Raz et al., 2014 [43]	17 drug naïve ADHD (10 males), no other neuropsychiatric comorbidities. Age range: 6–16 years (ALA group: 11.1 ± 3.00 years; Placebo group: 10.9 ± 2.30 years).	Two months DBRPCT Nine subjects taking Alpha-Linolenic Acid (ALA). Eight subjects taking Placebo.	ALA: 1 g/day. Placebo: similar to ALA.	MOXO-CPT: standardized computerized continuous performance test designed to evaluate ADHD-related symptoms. Four performance indices: attention, timing, impulsivity, and hyperactivity. CPRS-R: parent-rated behavioral indexes. CTRS-R: teacher-rated behavioral indexes.	No significant between-group difference was found in the changes of the various measures of ADHD symptoms throughout the study period.
Gustafsson et al., 2010 [44]	82 ADHD (number of males unspecified), no other neuropsychiatric comorbidities. Age range: 7–12 years (no mean age declared).	15 weeks DBRCT 40 subjects taking Omega 3. 42 subjects taking Placebo.	Omega 3: EPA 500 mg+ DHA 2.7 mg/day. Active capsules also contained 10 mg Vitamin E. Placebo: similar to Omega 3.	CPRS-R: parent-rated behavioral indexes. CTRS-R: teacher-rated behavioral indexes.	CTRS-inattention / cognitive subscale: children in supplementation group showed significant amelioration effect. CTRS total score: -48% of the children receiving supplementation vs. 9% of placebo improved ≥25%. -Among the less hyperactive ⁄ impulsive children, 36% of the ones receiving supplementation vs. 18% receiving placebo improved ≥25%. -Among the more hyperactive ⁄ impulsive children, 8 ⁄ 13 receiving supplementation vs. 1 ⁄ 9 receiving placebo improved ≥25%.
Hariri et al., 2012 [45]	103 ADHD (74 males), no other neuropsychiatric comorbidities. Age-range: 6–11. (Omega 3 group: 7.90 ± 1.53 years; Placebo group: 7.90 ± 1.45 years).	15 week DBRPCT 53 subjects taking Omega 3. 50 subjects taking Placebo.	Omega 3: EPA 635 mg+ DHA 195 mg/day day. Placebo: similar to Omega 3.	ASQ-P: parent-rated behavioral indexes.	ASQ-P: children in EPA + DHA group showed significant improvement.
Manor et al., 2012 [46]	147 ADHD (104 males), no other neuropsychiatric comorbidities. Age-range: 6–13 years (Supplementation group: 9.2 ± 2.0 years; Placebo group: 9.2 ± 1.8 years).	15 weeks DBRPCT + 15 weeks OL 100 subjects taking Phosphatidylserine (PS) + -Omega 3. 47 subjects taking Placebo.	PS: 300 mg/day. Omega 3: 120 mg/day (EPA/DHA ratio of 2:1). Placebo: similar to supplementation.	CPRS-R: parent-rated behavioral indexes. CTRS-R: teacher-rated behavioral indexes. SDQ: parent-rated emotional and behavioral indexes. CHQ: parent-rated measure of quality of life.	CPRS-R: -Significant reduction in the Global restless/impulsive subscale in supplementation group -Children with more severe symptomatology revealed a significant reduction in the ADHD-Index and hyperactive components. CPRS-R and CTRS-R: Children that switched to supplementation group from placebo showed a significant reduction in subscales severity scores compared to baseline. CHQ: significant improvement in Parent impact-emotional (PE) subscale in supplementation group.
Matsudaira et al., 2015 [47]	76 ADHD (76 males), no other neuropsychiatric comorbidities. Age-range: 12–16 years (LC-PUFA group: 13.7 ± 1.2 years; Placebo group: 13.7 ± 1.1 years).	Three months DBRPCT 38 subjects taking LC-PUFA 38 subjects taking Placebo.	LC-PUFA: EPA 558 mg + DHA 174 mg + CLA 60 mg + vitamin E 9.6 mg/day. Placebo: similar to LC-PUFA.	CTRS-R: teacher-rated behavioral indexes (specifically, the authors considered the ADHD index).	No between-group difference was found in the changes of the various measures of ADHD symptoms throughout the study period.
Milte et al., 2012 [48]	87 ADHD or parent-rated symptoms higher than the 90th percentile on the CPRS-R (67 males), including parent-reported learning difficulties. Age range: 6–13 years (EPA-rich group: 8.77 ± 1.76 years; DHA-rich group: 8.89 ± 1.60 years; LA-rich group: 9.14 ± 2.03 years).	Four months RCT 30 subjects taking EPA-rich oil. 28 subjects taking DHA-rich oil. 29 subjects taking LA oil.	EPA-rich oil: EPA 1109 mg+ DHA 108 mg/day. DHA-rich oil: DHA 1032 mg + EPA 264 mg/day. LA oil: 1467 mg/day. Each capsule also contained low concentration of vitamin E.	WIAT-III word reading and spelling subtests: literacy assessment. WISC-III vocabulary subtest: literacy assessment. CPRS-R: parent-rated behavioral indexes. Abbreviated test of everyday attention for children: attention assessment. Computerized go/no-go task: inhibition assessment.	No between-group or within-group difference was found in the changes of the various measures of symptoms throughout the study period. In a subgroup of 17 children with learning difficulties an increased erythrocyte DHA was more strongly associated with improved word reading (*r* = 0.683), improved spelling (*r* = 0.556), an improved ability to divide attention (*r* = 0.676), and lower parent ratings of oppositional behavior (*r* = 0.777), hyperactivity (*r* = 0.702), restlessness (*r* = 0.705), and overall ADHD symptoms (*r* = 0.665).
Milte et al., 2015 [49]	87 ADHD or parent-rated symptoms higher than the 90th percentile on the CPRS-R (67 males), including parent-reported learning difficulties. Age range: 6–13 years (8.91 ± 1.73 years).	12 months Three-way, crossover clinical trial Group 1: EPA→DHA→LA Group 2: DHA→LA→EPA Group 3: LA→EPA→DHA	EPA-rich oil: EPA 1109 mg+ DHA 108 mg/day. DHA-rich oil: DHA 1032 mg + EPA 264 mg/day. LA oil: 1467 mg/day. Each capsule also contained low concentration of vitamin E.	WIAT-III word reading and spelling subtests: literacy assessment. WISC-III vocabulary subtest: literacy assessment. CPRS-R: parent-rated behavioral indexes. Abbreviated Test of Everyday Attention for Children: attention assessment. Computerized go/no-go task: inhibition assessment.	No between-group or within-group difference was found in the changes of the various measures of symptoms throughout the study period. An increased proportion of erythrocyte EPA + DHA was associated with improved spelling (*r* = 0.365) and attention (*r* = −0.540) and reduced oppositional behavior (*r* = −0.301), hyperactivity (*r* = −0.310), cognitive problems (*r* = −0.326), DSM-IV hyperactivity (*r* = −0.270) and DSM-IV inattention (*r* = −0.343).
Mohammadzadeh et al., 2019 [50]	60 ADHD (49 males), no other neuropsychiatric comorbidities. Age range: 6–12 years (PUFA group: 8.20 ± 1.72 years; Placebo Group:7.7 ± 1.65 years).	Eight weeks DBRCT 31 subjects taking MPH + PUFA. 29 subjects taking MPH + Placebo.	Omega 3: EPA 180 mg + DHA 120 mg/day (from second week 2 times a day). Placebo: similar to Omega 3. MPH: 10 mg/day (in 2 doses); 20–30 mg/kg/day (in 2 doses) from second week.	ADHD-RS-IV parent rated	No significant effect was found.
Perera et al., 2012 [51]	94 ADHD (69 males), including ODD, CD, SLD and tics comorbidities. Age range: 6–12 years (Omega 3 group: 9.4 ± 1.5 years; Placebo group: 9.2 ± 1.5 years).	Six months DBRPCT 48 subjects * taking Omega 3/6. 46 subjects* taking Placebo. *subjects took MPH before trial.	Omega capsules: Omega 3 296.37 mg+ Omega 6 180.75 mg (ratio 1.6:1). Placebo: similar to Omega 3/6.	Parent-rated checklist assessing the following domains: aggressiveness, restlessness, inattention, distractibility, easy anger, impulsiveness, fighting, cooperation, completing work, wait for turn, academic performance. Total score range: 11–33.	Significant reduction in severity score in treatment group compared to placebo in the following measures: aggressiveness, restlessness, completing work, and academic performance, inattention, impulsiveness, and cooperation with parents and teachers.
Rodríguez et al., 2020 [52]	66 ADHD (47 males), including SLD, Anxiety, Tourette’s syndrome, psychomotor/behavioral problems comorbidities. Age range: 6–18 years (11.7 ± 3.1 years).	Six months DBRPCT 32 subjects * taking PUFA 34 subjects * taking Placebo * psychostimulant medication was allowed	PUFA sachet: DHA 1000 mg+ EPA 90 mg+ DPA 150 mg + vitamin E 4.5 mg+ carbohydrates 0.94 g (1 sachet/day in subjects ≤32 kg; 2 sachets/day in subjects >32 kg). Placebo: similar to PUFA sachet.	CPRS: parent-rated behavioral indexes. EDAH scale: parent-rated behavioral indexes. AULA Nesplora virtual test: test of attentional processes, impulsivity and motor activity. D2-test: paper and pencil test for selective and sustained attention.	CPRS: Significant within-group time effect: supplementation group showed improvement in behavioral variables; placebo group showed a behavioral symptomatology worsening. EDAH scale: Significant time x treatment interaction effect: supplementation group in post – treatment condition showed better behavioral indexes compared to placebo group. AULA Nesplora virtual test: Non-significant trend of higher amelioration in supplementation group. d-2 test: significant within-group time effect: both study groups showed improvements in cognitive variables.
Widenhorn-Müller et al., 2014 [53]	95 ADHD (74 males), no other neuropsychiatric comorbidities. Age range: 6–12 years (Omega 3 group: 8.90 ± 1.48 years; Placebo group: 8.92 ± 1.24 years).	Four months DBRPCT 46 subjects taking Omega 3. 49 subjects taking Placebo.	Omega 3: EPA 600 mg + DHA 120 mg/day. Active capsules also contained 15 mg Vitamin E. Placebo: similar to Omega 3.	HAWIK-IV: General cognitive ability, working memory, speed of information processing. DIS-YPS-II: parent-and teacher-rated questionnaires corresponding to the ICD-10 and DSM-IV diagnostic criteria for ADHD. CBCL: parent-rated questionnaire addressing behavioral and emotional problems. TRF: teacher-rated questionnaire addressing behavioral and emotional problems and academic performance. KITAP and TAP: computerized test batteries as measures for attentional performance.	HAWIK-IV: significant time x treatment interaction: supplementation group showed an improvement in working memory function compared to placebo – taking group. Improved working memory correlated with increased erythrocyte EPA and DHA and decreased AA.
**(b)**					
**Authors, Year**	**Sample and Age**	**Methodology and Durations**	**Daily Doses**	**Outcome Measures**	**Main Results**
Abbasi et al., 2011 [15]	40 ADHD (28 males), no other neuropsychiatric comorbidities. Age range: 7–13 years (ALC group:8.84 ± 2.03 years; Placebo group: 8.36 ± 1.53 years).	Six weeks DBRPCT 19 subjects taking MPH + Acetyl-L-carnitine (ALC). 19 subjects taking MPH + Placebo.	MPH week 1: 10 mg/day. MPH week 2: 20 mg/day. MPH week 3: 30 mg/day. ALC: 500–1500 mg/kg/day. Placebo + MPH: 20–30 mg/day/Kg.	Teacher and Parent rated ADHD-RS-IV.	Teacher and Parent rated ADHD-RS-IV: no significant between-groups outcome results. However, those in the ALC group experienced fewer adverse events than the placebo group regarding headaches and irritability.
Ghajar et al., 2018 [17]	50 ADHD (40 males), not excluding ODD. Age range: 6–17 years (l-carnosine group: 9.12 ± 2.18 years; Placebo group: 8.28 ± 1.59 years).	Eight weeks DBRPCT 25 subjects taking MPH + l-carnosine. 25 subjects taking MPH + Placebo.	MPH: 0.5–1.5 mg/kg/day. Week 1:10 mg/day. Week 2-end: 20 mg/day. Subjects >30 kg: 30 mg/day. l-carnosine: 800 mg/day. Placebo: 800 mg/day.	Primary outcome: Parent rated ADHD-RS-IV. Secondary outcome: Teacher rated ADHD-RS-IV. Both rated at baseline and at weeks four and eight.	Primary outcome: Parent rated ADHD-RS-IV. Significant time x treatment interaction effect both at weeks 4 and 8: MPH + supplementation group showed greater improvements compared to MPH + placebo group. Secondary outcome: Teacher rated ADHD-RS-IV. No significant time x treatment interaction effect.
Hirayama et al., 2013 [21]	36 ADHD (34 males), no other neuropsychiatric comorbidities. Age range: 4–14 years (Phosphatidylserine group: 9.1 ± 1.7 years; Placebo: 8.7 ± 3.0 years).	Eight weeks DBRPCT 19 subjects taking Phosphatidylserine (PS). 17 subjects taking Placebo.	PS: 100 mg/day. Placebo: 100 mg/day.	ADHD diagnostic criteria of DSM-IV-TR. WISC-III (Digit Span Test). go/no-go experiment.	Significant treatment effect: supplementation group showed post-treatment improvements compared to pre-treatment condition in ADHD, AD and HD symptoms (DSM-IV-TR), short-term auditory memory (WISC-III) and total number of errors in the reverse differentiation test (inattention – impulsivity), as well as total inattention errors and total errors over time (Go/No-Go). No significant differences were observed in other measurements and in the placebo group.
**(c)**					
**Authors, Year**	**Sample and Age**	**Methodology and Durations**	**Daily Doses**	**Outcome Measures**	**Main Results**
Arnold et al., 2011 [54]	52 ADHD (43 males), ODD, CD comorbidities. Age range: 6–14 years (Zinc group 1: 9.61 ± 3.36 years; Zinc group 2: 8.89 ± 2.31 years; Placebo group: 10.24 ± 2.69 years).	13 weeks (8 + 5) DBRPCT + 8 weeks OL Phase 1 28 subjects taking Zinc glycinate (group 1 and 2). 24 subjects taking Placebo. Phase 2/3 28 subjects taking Zinc glycinate + d-amphetamine. 24 subjects taking Placebo + d-amphetamine.	Zinc glycinate group 1: 15 mg/day (20 subjects). Zinc glycinate group 2: 15 mg/2 times day (8 subjects). AMPH: 25 kg: 5 mg/day; 25–45 kg: 10 mg/day; >45 kg: 15 mg/day. Placebo: similar to Zinc glycinate.	Parent and Teacher rated SNAP IV: checklist of DSM-IV ADHD symptoms. CPRS-R: parent-rated behavioral indexes. Short-term recognition memory task. Continuous performance task 11. Seat activity using a ‘‘wiggle’’ seat. CGI-S and CGI-I: assessment of severity and improvement as reported by clinician. Parent and child children’s depression inventory.	Phase 1: Parent and teacher rating showed no consistent tendency of superiority of zinc over placebo. Neuropsychological cognitive motor results are inconsistent, although a bit more favorable to zinc. *Phase 2/3:* Optimal mg/kg AMPH dose with in zinc group 2 was 37% lower than with placebo.
Noorazar et al., 2020 [55]	60 ADHD (48 males), no other neuropsychiatric comorbidities. Age range: 7–12 years (Control group: 9.30 ± 1.38 years; Zinc group: 8.87 ± 1.97 years).	Six weeks DBRPCT 30 subjects taking MPH+ Zinc sulfate syrup. 30 subjects taking MPH + Placebo.	MPH: 0.5–1 mg/kg/day. Zinc sulfate syrup: 10 mg/day Placebo: 10 mg/day.	CPRS-R: parent-rated behavioral indexes.	CPRS-R. Significant time x treatment interaction effect: MPH + supplementation group showed greater improvements compared to MPH + placebo group in inattention score.
Salehi et al., 2016 [56]	150 ADHD (111 males), no other neuropsychiatric comorbidities. Age range: 6–15 years (Control group: 9.12 ± 2.2 years; Omega 3 group: 8.6 ± 1.7 years; Zinc group: 9.5 ± 2.5 years).	Eight weeks DBRCT 50 subjects taking MPH+ Placebo. 50 subjects taking MPH+ Omega 3. 50 subjects taking MPH+ Zinc sulfate.	MPH: 0 mg/day for subjects <20 kg; 10 mg/2 times a day > 20 kg. Omega 3: 100 mg EPA for subjects <25 kg; 200 mg for subjects 26–35 kg;400 mg for subjects >35 kg/day. Zinc sulfate: 22 mg/day.	CPRS-R (parent-rated behavioral indexes) and CTRS-R (teacher-rated behavioral indexes) every 2 weeks.	CPRS-R and CTRS-R: Significant time x treatment interaction effects in children with attention deficit disorder subtype: - zinc group showed greater improvements compared to placebo group; - Omega 3 group showed greater improvements compared to the zinc group.
Dehbokri et al., 2019 [57]	96 ADHD (80 males), no other neuropsychiatric comorbidities. Age range: 2–18 years (Vitamin D group: 9.76 ± 2.38 years; Placebo group: 8.58 ± 2.02 years).	Six weeks DBRCT 51 subjects taking MPH + Vitamin D. 45 subjects taking MPH + Placebo.	MPH: unspecified. Vitamin D3: 50,000 IU/week. Placebo: similar to Vitamin D3.	CPRS-R: parent-rated behavioral indexes.	CPRS-R: Significant time x treatment interaction effect: supplementation group showed a significant decrease in hyperactivity, impulsivity and attention problems compared to placebo group. These results improved considerably in patients with insufficient levels of Vitamin D at baseline.
Elshorbagy et al., 2018 [58]	35 ADHD with vitamin D deficiency, including ODD, SLD comorbidities (number of males unspecified). Age range: 7–14 years (9.3 ± 2.6 years).	12 weeks Case-Control Prospective Interventional Study 16 subjects taking MPH + Vitamin D. 19 subjects taking MPH + Placebo.	MPH: 0.3–1 mg/kg/3 times a day. Vitamin D3: 3000 IU/day. Placebo: similar to Vitamin D3.	CPRS-R: parent-rated behavioral indexes. Weekly Parent Ratings Behaviour.	CPRS-R: Significant time x treatment interaction effect: ADHD who received vitamin D showed a significant improvement in conceptual level, inattention, opposition, hyperactivity and impulsivity compared with placebo group.
Mohammadpour et al., 2018 [25]	62 ADHD (46 males), no other neuropsychiatric comorbidities. Age range: 5–12 years (Vitamin D group: 7.70 ± 1.77 years; Placebo group: 8.03 ± 1.44 years).	Eight weeks DBRPCT 25 subjects taking MPH + Vitamin D. 29 subjects taking MPH + Placebo.	MPH: 0.3–1 mg/kg/3 times a day. Vitamin D tablets: 2000 IU/day. Placebo: similar to Vitamin D.	WPREMB: parent-rated morning and evening behavioural indexes. Parent rated ADHD-RS-IV. CPRS-R: parent-rated behavioral indexes.	WPREMB: Significant time x treatment interaction effect: ADHD who received vitamin D showed a significant improvement in total score and evening symptoms compared to placebo group. ADHD-RS: Significant within-group time effect: ADHD who received vitamin D showed an improvement in total score.
**(d)**					
**Authors, year**	**Sample and age**	**Methodology and durations**	**Daily doses**	**Outcome measures**	**Main results**
Borlase et al., 2019 [59]	27 ADHD drug naïve (27 males), excluding only ASD and epilepsy comorbidities. Age range: 7–12 (Micronutrients group: 10.75 ± 1.50 years; Placebo group: 10.17 ± 1.36 years).	10 weeks DBRPCT 13 subjects taking Micronutrients (DEN Formula). 14 subjects taking Placebo.	Micronutrients (comprising 13 vitamins, 17 minerals, 4 amino acids): titration over a week up to 12 capsules/day (in 3 doses). If there was no clinical response after four weeks, subjects could choose to take 15 pills/day. Placebo: similar to micronutrients.	CGI-I: assessment of improvement as reported by clinician. Clinician-rated ADHD-RS-IV. CPRS-R: parent-rated behavioral indexes. CTRS-R: teacher-rated behavioral indexes. Magnetic Resonance Imaging (MRI)	Questionnaires: Significant advantage of supplementation group over placebo for general functioning, emotional dysregulation, aggression and inattention. MRI: No significant between-groups differences. In the treatment group there was a non-significant trend for: - decreased choline in the striatum; - decreased glutamate in the prefrontal cortex; - increased grey matter in the anterior thalamus; - increased white matter in the fornix; - improved network integrity of the default mode network, dorsal attention network and frontal executive network.
Hemamy et al., 2020 [60]	66 ADHD (46 males), no other neuropsychiatric comorbidities. Age range: 6–12 years (Vitamin D group: 9.06 ± 1.76 years; Placebos group: 9.15 ± 1.46 years).	Eight weeks DBRCT 33 subjects taking MPH + Vitamin D + Magnesium. 33 subjects taking MPH + Placebos.	MPH: unspecified. Vitamin D: 50,000 IU/week. Mg: 6 mg/kg/day. Placebo: similar to Vitamin D or Mg.	CPRS-R: parent-rated behavioral indexes.	CPRS-R: Significant time x treatment interaction effect: ADHD who received supplementation showed a significant improvement in conduct problem score, social problem and anxiety score compared to placebo group.
Rucklidge et al., 2018 [61]	93 ADHD (69 males), excluding only ASD and epilepsy comorbidities. Age range: 7–12 years (Micronutrients group: 10.06 ± 1.56 years; Placebo group: 9.43 ± 1.53 years).	10 weeks FBRPCT 47 subjects taking Micronutrients. 46 subjects taking Placebo.	Micronutrients: 3–12/15 capsule/day divided into 3 doses (it contains 13 vitamins, 17 minerals, 4 amino acids). Placebo: similar to micronutrients.	CGI-I and C-GAS: assessment of severity and improvement as reported by clinician. Clinician-rated ADHD-RS-IV. CPRS-R: parent-rated behavioral indexes. CTRS-R: teacher-rated behavioral indexes. SDQ: parent- and teacher-rated emotional and behavioral indexes. BRIEF: teacher-rated behavioural measures of executive skills in everyday environment.	CGI-I: the number of responders in supplementation group was 20 (51%) versus 11 (27%) on placebo. Clinician-rated ADHD-RS-IV. Improvement in inattention and hyperactivity symptoms, aggression, emotional dysregulation, conduct problem and problem behaviour in ADHD who received supplementation compared with placebo group.
Darling et al., 2019 [62]	84 ADHD no drug naïve (62 males). See Rucklidge 2018. 43 subjects from Micronutrients group; 41 subjects from Placebo group.	Naturalistic Follow-up Study after 12 month post-baseline 19 subjects stayed on trial micronutrients. 21 subjects switched to medications. 35 subjects stopped all treatments. Nine subjects mixed micronutrients and medications. 27/84 subjects added psychological/ other intervention.	Micronutrients: 8–15 capsule/day (it contains 13 vitamins, 17 minerals, 4 amino acids).	CPRS-R: parent-rated behavioral indexes. SDQ: parent-rated emotional and behavioral indexes. Parent-rated CMRS for a measure of emotion dysregulation. Parent-rated SCARED-R for a measure of anxiety symptoms. Eating Behaviour Questionnaire. Acceptability of Treatment questionnaire.	More of those who stayed on supplementation (84%) were identified as ‘‘Much’’ or ‘‘Very Much’’ improved overall relative to baseline functioning, compared to 50% of those who switched to psychiatric medications and 21% of those who discontinued treatment. 79% of those still taking micronutrients, 42% of those using medications, and 23% of those who discontinued treatment were considered remitters based on parent-reported ADHD.
Rucklidge et al., 2019 [63]	71 ADHD (55 males). See Rucklidge 2018. Age range: 7–12 (9.7 ± 1.5 years).	Data from Rucklidge (2018) +10 weeks OL 40 subjects from RCT phase (taking micronutrients). 31 subjects from OL phase (taking micronutrients).	Micronutrients: 3–12/15 capsule/day divided into 3 doses (it contains 13 vitamins, 17 minerals, 4 amino acids). No Placebo.	Clinician-rated ADHD-RS-IV. CGI-I and C-GAS: assessment of severity and improvement as reported by clinician. CPRS-R: parent-rated behavioral indexes. Parent-rated CMRS for a measure of emotion dysregulation. SDQ: parent-rated emotional and behavioral indexes.	Varying predictors were found across outcomes: lower pre-treatment folate and B12 levels, being female, greater severity of symptoms and co-occurring disorders in pre-treatment condition, more pregnancy complications and fewer birth problems were identified as possible predictors of greater improvement for outcome measures.
**(e)**					
**Authors, year**	**Sample and age**	**Methodology and durations**	**Daily doses**	**Outcome measures**	**Main results**
Katz et al., 2010 [64]	92 ADHD (92 males), no other neuropsychiatric comorbidities. Age range: 6–12 years (CHP group:9.82± 1.56 years; Placebo group: 9.36 ± 1.97 years).	Four months DBRPCT 73 subjects taking Compound Herbal Preparation (CHP). 19 subjects taking Placebo.	CHP: 3 mL three times daily, before meals, diluted in 50 to 60 mL of water. Placebo: similar to CHP.	TOVA task to measure attention Daily side effect questionnaire	TOVA task: Significant within-group time effect: supplementation group showed significant improvement in the 4 subscales and overall scores, compared with no improvement in the control group. No serious side effects were reported.
Ko et al., 2014 [65]	70 ADHD/ADHD NOS (44 males), no other neuropsychiatric comorbidities. Age range: 6–15 (KRG group = 10.94 ± 2.26 years; Placebo group = 10.86 ± 2.41 years).	Eight weeks DBRPCT 33 subjects taking Korean Red Ginseng (KRG). 37 subjects taking Placebo.	KRG: 1g (extract/pouch) twice a day. Placebo dose: one pouch twice a day.	Primary outcome: DSM-IV criteria for inattention and hyperactivity scale scores. Secondary outcomes: QEEG TBR: EEG theta/beta ratio. Salivary cortisol. DHEA levels.	Primary outcome: Active treatment significantly improved the inattention scores and hyperactivity scores. Secondary outcomes: - Supplementation group showed a significantly decrease in QEEG TBR. - No significant effect of supplementation on cortisol and DHEA levels. - No serious adverse reactions to KRG.
Li et al., 2011 [66]	72 ADHD (47 males), no other neuropsychiatric comorbidities. Age range: 6–13 years (NDG group: 9.3 ± 1.8 years; MPH group: 9.2 ± 2.2 years).	Eight weeks DBRCT 36 subjects taking MPH. 36 subjects taking Ningdong granule (NDG).	MPH: 1 mg/kg/day. NDG: 5 mg/kg/day.	Teacher and Parent ADHD-RS to measure behavior. Blood levels of dopamine (DA) and homovanillic acid (HVA) Side effect questionnaire	Teacher and parent ADHD-RS: Scores were reduced from baseline to week 8 in both groups, but there were no significant differences between NDG and MPH groups. MPH group had more side effects than NDG group (significant effect only in hypersomnia). DA levels showed no significant change during the study. HVA in supplementation group was higher at the end of the research, but there was no significant difference between two groups. HVA increasing was associated with improved scores of Teacher and Parent ADHD-RS.
Motaharifard et al., 2019 [67]	50 ADHD (33 males), no other neuropsychiatric comorbidities. Age range: 6–14 years (MPH group: 7.5 ± 1.5 years; Sweet almond group: 6.6 ± 1.0 years).	Eight weeks TBRCT 25 subjects taking MPH+ Placebo syrup. 25 subjects taking Placebo tablet + Sweet almond syrup.	MPH: week 1–5 mg tablet twice daily; from week 2–10 mg tablet twice daily; subjects >30 kg received a 10 mg tablet thrice daily from the third week. Placebo syrup: 5 cc/3 times a day. Sweet almond syrup: 5 cc/3 times a day. Placebo tablet: 5 mg twice daily.	Teacher and Parent ADHD-RS to measure behavior, every 2 weeks. Side effects checklist.	Teacher and Parent ADHD-RS: No significant differences were observed between the two groups. Both groups exhibited a similar declining linear trend in ADHD symptoms over time. Sweet almond syrup had less side effects (only increased appetite).
Salehi et al., 2010 [68]	50 ADHD (39 males), no other neuropsychiatric comorbidities. Age range: 6–14 years (G. biloba group: 9.12 ± 1.61 years; MPH group: 9.61 ± 2.26 years).	Six weeks DBRCT 25 subjects taking Ginkgo biloba. 25 subjects taking MPH.	G. biloba: 80–120 mg/day/kg (80 mg/day for <30 kg and 120 mg/day for >30 kg). MPH: 20–30 mg/day/kg (20 mg/day for <30 kg and 30 mg/day for >30 kg).	Primary outcomes: Parent and Teacher ADHD-RS to measure behavior. Secondary outcomes: Side effect checklist. Physiological parameters.	Parent and Teacher ADHD-RS: Supplementation was less effective than MPH. The difference between supplementation and MPH groups in the frequency of side effects was not significant, except for more frequent decreased appetite, headache and insomnia in the MPH group.
Shakibaei et al., 2015 [69]	60 ADHD (39 males), no other neuropsychiatric comorbidities. Age range: 6–12 years (G. biloba group: 7.83 ± 1.21; Placebo group: 8.41 ± 1.40 years).	Six weeks DBRPCT 31 subjects taking MPH* + Ginkgo biloba. 29 subjects taking MPH* + Placebo. *subjects took MPH before the trial.	MPH: 20 mg/day (10 mg/b.i.d) for subjects <30 kg; 30 mg/day (10 mg/t d s) for subjects >30 kg. G. biloba: 80 mg/day (40 mg/b.i.d) for subjects <30 kg; 120 mg/day (40 mg/t d s) for subjects >30 kg. Placebo: similar to G. biloba tablet.	Primary outcomes: Parent and Teacher ADHD-RS to measure behavior. Secondary outcomes: C-GAS: assessment of symptoms severity as reported by clinician. Physiological parameters. Side effect checklist.	Parent and Teacher ADHD-RS: -A significant improvement was found in inattention score and parent total rating score in supplementation group. - Response rate was higher in supplementation group compared to placebo based only on parent rating. C-GAS: No significant between-group difference after treatment. No between-group significant difference in side effects.
Tan et al., 2016 [70]	146 ADHD (124 males), excluding syndromes, inborn errors of metabolism, brain lesions, chronic liver disease, anticoagulant/antiplatelet drugs. Age range: 6–12 years (TRF^a^ group: 9.4 ± 1.9; Placebo group: 9.4 ± 1.7).	One month run in with placebo + 6 months RPCT 73 subjects taking TRF^a^ (43 subjects taking medication). 73 subjects taking Placebo (35 subjects taking medication).	TRF^a^ capsules: 200 mg/day. Placebo: similar to TRF ^a^.	VAPRS: parent-rated ADHD symptoms. VATRS: teacher-rated ADHD symptoms. Side effects questionnaire. Tocotrienol levels (blood exams).	VAPRS: significant improvement in both groups. VATRS: improvement in TRF^a^ group but not statistically significant. Side effects: non-significant differences between groups. Tocotrienol levels: higher levels in TRF^a^ group and significant correlation with the change in VAPRS.
**(f)**					
**Authors, year**	**Sample and age**	**Methodology and durations**	**Daily doses**	**Outcome measures**	**Main results**
Pärtty et al., 2015 [71]	75 TD children (40 males). Age range at RCT: 0–6 months after birth. Age range at follow-up: 13 years. Diagnoses at 13 years: 3 ADHD; 1 AS; 2 ADHD + AS.	Six months after birth DBPCRT+ Follow-up at 13 years 40 subjects taking *Lactobacillus rhamnosus* GG. 35 subjects taking Placebo.	*Lactobacillus rhamnosus* GG: 1 × 10^10^ colony-forming units\day for 4 weeks before delivery + for 6 months after delivery.	ICD-10 diagnostic criteria for diagnoses of ADHD or Asperger syndrome (AS) filled in at follow-up. Gut microbiota: in situ hybridization (FISH) and qPCR and blood group secretor type.	ADHD or AS was diagnosed in 6/35 (17.1%) children in the placebo and none in the probiotic group (*p* = 0.008). The mean numbers of *Bifidobacterium* species bacteria in feces during the first 6 months of life was lower in children with ADHD or AS log cells/g than in healthy children.

Note: AA: arachidonic acid; AD: attention deficit; ADHD: attention deficit hyperactivity disorder; ADHD NOS: attention deficit hyperactivity disorder not otherwise specified; ADHD-RS: attention deficit hyperactivity disorder rating scale; AL: alpha-linolenic acid; ALC: Acetyl-L-carnitine; AMPH: d-amphetamine; AS: Asperger syndrome; ASD: autism spectrum disorder; ASQ-P: Conners’ abbreviated questionnaires; ATX: atomoxetine; BRIEF: behavior rating inventory of executive function; CBCL: child behavior checklist; CD: conduct disorder; CGI-S/I: clinical global impression—severity scale/improvement scale; C-GAS: children’s global assessment scale; CGQ: child health questionnaire–parent form; CHQ: child health questionnaire; CHP: compound herbal preparation; CLA: conjugated linoleic acid; CPRS-R: revised Conners’ parent rating scale; CPT: continuous performance task; CTRS-R: revised Conners’ teacher rating scale; DA: dopamine; DHA: docosahexaenoic acid; DHEA: dehydroepiandrosterone; DBRCT: double blind randomized controlled/clinical trial; DBRPCT: double blind randomized placebo-controlled/clinical Trial; DEN: daily essential nutrients; DIS-YPS-II Diagnostik psychischer Störungen im Kindes-und Jugendalter; DPA: docosapentaenoic acid; DSM-IV: diagnostic and statistical manual of mental disorders (4th ed.); EPA: eicosapentaenoic acid; FBRPCT: fully blinded randomized placebo-controlled trial; GLA: gamma linoleic acid; HAWIK-IV: Hamburg Wechsler Intelligence Scales for Children–IV; HD: hyperactivity disorder; HVA: homovanillic acid; IU: international unit; KITAP Test-batterie zur Aufmerksamkeitsprüfung für Kinder; KRG: Korean red ginseng; LA: linoleic acid; M: males; MOAS: modified overt aggression scale; MPH: methylphenidate; MSVA: Magallanes scale of visual attention; (f)-MRI: (functional) magnetic resonance imaging; NDG: ningdong granule; ODD: oppositional defiant disorder; OL: open label; (LC)-PUFAs: (long-chain) polyunsaturated fatty acids; PS: phosphatidylserine; QEEG TBR: quantitative electroencephalography theta/beta ratio; RPCT: randomized placebo-controlled trial; RPCCT: randomized placebo-controlled crossover trial; RPT: randomized pilot trial; RT: randomized trial; SCARED: screen for child anxiety related emotional disorders; SDQ: strengths and difficulties questionnaire; SLD: specific learning disorder; SNAP: Swanson, Nolan, and Pelham rating scale; TAP Testbatterie zur Aufmerksamkeitsprüfung; TD: typically developing; TOVA: test of variable of attention; TRF: teacher’s report form; TRF^a^: tocotrienol-rich fractions; VAPRS: Vanderbilt ADHD parent rating scale (NICHQ); VATRS: Vanderbilt ADHD teacher rating scale (NICHQ); WIAT-III: Wechsler individual achievement test; WISC: Wechsler intelligence scale for children; WPREMB: weekly parent ratings of evening and morning behavior.

**Table 2:** Summary of sources of information that were considered in the reviewed studies.

	Self Rating Scales	Parent Rating Scales	Teacher Rating Scales	Clinician Rated Scales	Psychometric Tests	Computerized Tasks	Neurophysiological Measures
PUFAs	[39,40]	[34,35,36,37,38,39,40,41,42,43,44,45,46,48,49,50,51,52,53]	[37,39,43,44,46,47,53]	[36,41]	[40,42,48,49,52,53]	[38,39,42,43,48,49,52] [53]	[38]
Peptides and amino acids derivatives		[15,17]	[15,17]	[21]	[21]	[21]	
Single micronutrient		[54,55,56,57,58,25]	[54,56]	[54]	[54]	[54]	
Micronutrients mix		[59,60,61,62,63]	[59,61]	[59,61,63]			[59]
Plant / herbal extracts	[64,66,67,68,69,70]	[64,66,67,68,69,70]	[66,67,68,69,70]	[64,65]		[64]	[65]
Probiotics				[71]			
